# Experimental evidence for heterospecific alarm signal recognition via associative learning in wild capuchin monkeys

**DOI:** 10.1007/s10071-019-01264-3

**Published:** 2019-05-08

**Authors:** Brandon C. Wheeler, Martin Fahy, Barbara Tiddi

**Affiliations:** 1 School of Anthropology and Conservation, University of Kent, Canterbury, CT2 7NR UK; 2 Cognitive Ethology Laboratory, German Primate Center, 37077 Göttingen, Germany; 3 Proyecto Caí, Iguazú National Park, Puerto Iguazú, Misiones Argentina; 4 Department of Behavioral Ecology, Johann-Friedrich-Blumenbach Institute for Zoology and Anthropology, Georg-August University, 37077 Göttingen, Germany

**Keywords:** Alarm calls, Anti-predator behaviour, Associative learning, Communication, Information, New World primates

## Abstract

Many vertebrate taxa respond to heterospecific alarm calls with anti-predator behaviours. While it is unclear how apparent recognition is achieved, learned associations between the occurrence of the call and the presence of a predator are considered the most likely explanation. Conclusive evidence that this behaviour is indeed underpinned by learning, however, is scarce. This study tested whether wild black capuchin monkeys (*Sapajus nigritus*) learn to associate novel sounds with predators through a two-phase field experiment. During an initial training phase, three study groups were each presented with a playback of one of the three novel sounds together with a simulated felid predator on four occasions over an 8- to 12-week period. This was followed by a test phase, wherein each of the three sounds was played back to individuals in all three groups, allowing each sound to serve as both a test stimulus for individuals trained with that sound, and a control stimulus for individuals trained with another sound. Antipredator responses were significantly stronger in response to test sounds than to controls. Limited observations suggest that antipredator responses persisted for at least 2 years without reinforcement of the predator–sound link. Additionally, responses to noisier sounds were typically stronger than were those to more tonal sounds, although the effect of sound type cannot be disentangled from potential effects of group. This study provides the strongest evidence to date that learning affects the responses of primates to sounds such as heterospecific alarm calls, and supports the contention that signals provide receivers with information.

**Electronic supplementary material:**

The online version of this article (10.1007/s10071-019-01264-3) contains supplementary material, which is available to authorized users.

## Introduction

Whether or not animal communication should be conceptualised as information transfer has long been a contentious issue (Dawkins and Krebs 1978; Rendall et al. 2009). While the issue initially focused on whether it would be adaptive for signallers to provide information to receivers (Dawkins and Krebs 1978; Krebs and Dawkins 1984), more recent debates have largely centred on whether the proximate mechanisms underlying the responses of signal receivers are in line with contentions that receivers interpret signals as informative (Rendall et al. 2009; Seyfarth et al. 2010). Recent critics of the informational perspective argue that receiver responses can largely be explained as unconscious nervous system-based reactions to the physical aspects of the signal, with natural selection shaping signal structure to elicit reactions that preferentially benefit the signal producer (Rendall et al. 2009). In contrast, proponents of the informational perspective point to studies that suggest that receiver responses to a given signal are driven by learned associations between the production of the signal and the co-occurrence of something salient to the receiver (Seyfarth et al. 2010; Wheeler and Fischer 2012), with a number of avian and mammalian taxa, particularly primates, argued to provide compelling evidence of such learning (Seyfarth and Cheney 2010; Fischer 2011). However, evidence that responses to signals are shaped by learning is largely indirect, with no direct evidence in nonhuman primates.

Some of the strongest evidence that associative learning shapes signal response in wild animals comes from studies of responses of various taxa to heterospecific alarm calls (Hauser 1988; Terborgh 1990; Rainey et al. 2004; Ito and Mori 2010; Magrath and Bennett 2012; see also Mitchell and McCormick 2013). It is often assumed that such responses are due to individuals of one species learning that the production of other species’ alarms is associated with the presence of a predator (Fischer 2011). However, it is also plausible that responses to the alarm calls of other species are innate, nervous system-based reactions to the physical features of the sound, given that alarm calls across taxa tend to be characterised by similar acoustic features (e.g., sudden onsets and pulses of energy) (Owren and Rendall 2001; Rendall et al. 2009). While most evidence points to a role of learning (Hauser 1988; Magrath et al. 2009; Magrath and Bennett 2012), the two hypotheses regarding the proximate basis for anti-predator responses to heterospecific alarms are not mutually exclusive (Owren and Rendall 2001; Fallow et al. 2013), and a role for acoustic features in driving responses has also received some support (Fallow et al. 2011, 2013). Despite the widespread evidence that responses to heterospecific alarm are shaped at least in part by learning, support has been largely indirect (Magrath et al. 2015a). More conclusive, direct evidence of learning is rare (Shriner 1999; Magrath et al. 2015b), and lacking completely for nonhuman primates.

Demonstration of a role of learning in signal perception among primates is important given that studies of communication in this taxon have been central in the debate regarding whether receivers in animal communication are informed by signals (Owren and Rendall 2001; Seyfarth et al. 2010). Robust capuchin monkeys (*Sapajus* spp., taxonomically synonymous with *Cebus apella*) in particular are ideal for testing the learning hypothesis as they have been shown to be proficient learners in a variety of contexts (Coelho et al. 2015), and are known to respond to the alarm calls of a number of sympatric prey taxa with anti-predator behaviours (Wheeler and Hammerschmidt 2013; Di Bitetti and Wheeler 2017). Here, we test whether the responses of black capuchins (*Sapajus nigritus*; synonymous with *C. apella nigritus*) to predator-associated signals may be based in part on learning. To do this, we first exposed three wild groups to a novel sound (a different sound for each group), while simultaneously presenting a felid predatory stimulus (visual decoys or playbacks of recorded predator calls) during a 2- to 3-month training phase. Following this, we conducted a test phase in which we played each of the three novel sounds to individuals in each group in the absence of any additional predatory stimulus. If responses to heterospecific alarm signals are indeed shaped by learning, we predicted that individuals would respond more strongly to the novel sounds that they heard in association with a predator during the training phase than to those that they did not hear in this context. We further examined whether the three novel sounds differed in the responses they elicited, and whether responses to conditioned stimuli weakened over time.

## Methods

### Study site and subjects

This study was conducted with wild black capuchin monkeys in Iguazú National Park (25°40′43″S, 54°26′57″W), part of the Upper Paraná Atlantic Forest in northeastern Argentina, from late May/early June through August in each year from 2011 to 2014. The park consists of 57,000 ha of protected, semi-deciduous subtropical rainforest with a dense understory. Black capuchins are medium-sized (2.5–3.5 kg), mostly arboreal primates that tend to inhabit the middle and lower canopy and forest understory (Fleagle 2013). Black capuchins at the site live in stable, cohesive multimale–multifemale groups that typically contain 7–30 individuals (Janson et al. 2012). This study was conducted with three capuchin groups: Rita group (12–18 individuals during the study period), Macuco group (22–24 individuals), and Spot group (14–21 individuals). Capuchins at the site face predation from a number of felid predators including ocelots (*Leopardus pardalis*), pumas (*Puma concolor*), and jaguars (*Panthera onca*), as well as at least two aerial predators (hawk eagles: *Spizaetus* spp.), while pit vipers pose a mortal but non-predatory threat (Janson et al. 2012). Like most primates, black capuchins produce different vocalisations in response to each of terrestrial and aerial threats (“hiccups” and “barks”, respectively) (Wheeler 2008, 2010). In addition, capuchins produce hiccups in response to the apparent alarm calls of a number of other understory-dwelling species that can be reasonably assumed to be preyed upon by the same felid predators that threaten capuchins (Azara’s agouti: *Dasyprocta azarae*; white-shouldered fire eyes: *Pyriglena leucoptera*; dusky legged guan; *Penelope obscura*) (Wheeler and Hammerschmidt 2013; Di Bitetti and Wheeler 2017).

### Experimental methods

**Fig. 1:**
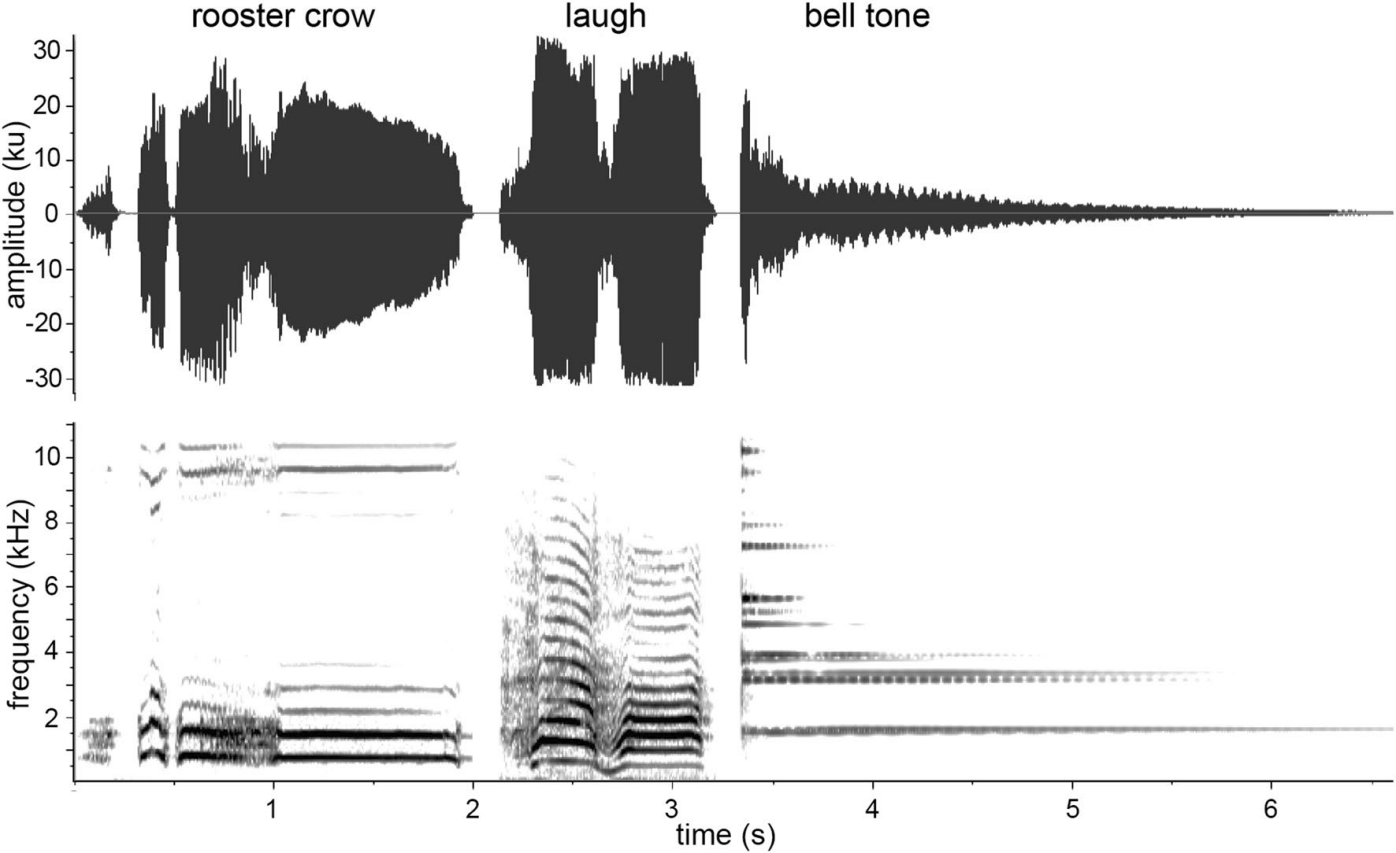
Spectrograms of the three novel stimuli paired with felid predator stimuli in this study. Spectrograms were made in Raven 1.5 Pro (Cornell Lab of Ornithology: http://www.birds.cornell.edu/raven) using a 256-point Hamming window with 50% overlap (3 dB bandwidth = 128 Hz), 256-point DFT, and a time and frequency measurement precision of 5.8 ms and 86.2 Hz, respectively

To determine if capuchins learn to associate novel sounds with the presence of predators, an initial 2- to 3-month training phase was conducted (in 2011 for the Rita group, and in 2012 for the Macuco and Spot groups), in which a visual or acoustic predator stimulus was presented to the monkeys simultaneously with a playback of a novel sound that is distinct from sounds that the monkeys might hear in other contexts. During the 3-month training period, each group was exposed to a predator–novel sound pairing on four occasions at 16–40-day intervals (mean ± SD 22 ± 7.3 days), with a different sound used with each group: a rooster’s (*Gallus gallus domesticus*) crow, a distinct human “laugh” (the signature, mocking laugh of the Nelson Muntz character from the television show *The Simpsons*), and a monotonous bell tone were used for the Rita, Macuco, and Spot groups, respectively (Fig. 1; sounds available for download in the Electronic Supplementary Material). Although pre-training playbacks would have been useful to demonstrate a change in behaviour following the training phase, we avoided this to strengthen the sound–predator link based on a small number of pairings. For each group, the visual stimulus (a decoy ocelot; see the Electronic Supplementary Material) was used on three of these occasions. To minimise habituation to the models, a puma’s vocalisation was used as the predatory stimulus during one training session. The puma call was recorded at the study site with a Sennheiser ME67/K6 shotgun microphone connected to a Marantz PMD-660 digital audio recorder, during a chance encounter with a vocalising puma. In the training phase, the predator’s call was played through a concealed Saul Mineroff Electronics Amplified Field Speaker connected to an Apple iPod Touch audio player at a sound pressure level of approximately 70–80 dB as measured 1 m from the speaker. For both types of predator stimulus, the novel sound was played through a RadioShack mini-amplified speaker (#277-1008) connected to an Apple iPod Touch audio player via a 10-m cable, at a sound pressure level of approximately 70–80 dB as measured 1 m from the sound source. The speaker was placed concealed on the ground hidden approximately 10 m from the predator stimulus. Both the mini-amplified speaker and the predator stimulus (decoy or speaker) were first positioned at least 50 m from the group in the direction of their movement and left stationary. In the case of the visual predator stimulus, the novel sound was played after the first individual in the group detected the model, which was invariably followed by the production of terrestrial predator alarm calls, the approach of additional group members, and an extended period of mobbing (Wheeler 2008, 2010). The novel sound was played approximately 60 s after the detection of the model and initiation of alarm calls by the capuchins, and was played five–seven times at approximately 60-s intervals, while the group engaged in mobbing and vigilance behaviours towards the model. If the group did not move away within 30 min of the initial detection, the decoy was covered with a tarp. In the case of the auditory predatory stimulus, the sound of the puma’s vocalisation was played when the front of the group was within 15–25 m of the speaker. Capuchins responded to this auditory stimulus by producing terrestrial predator alarm calls, approaching the speaker (presumably in an attempt to locate the predator), and becoming highly vigilant in the area around the playback. The puma’s call was played 15–20 times over 3–4 min. The novel sound was played through the second speaker five–eight times between and after calls from the puma.

To determine whether individuals in each group learned to associate their group’s novel sound with the presence of a predator, a test phase was conducted in which all three of the novel sounds were played back to subjects in each of the three study groups, but in the absence of any simulated predator. Each of the three novel sounds, thus, served as a test stimulus for individuals that heard that sound in conjunction with a simulated predator, and a control stimulus for those that never heard that sound in a predator-related context. A total of 18 test playbacks (five using the rooster, seven using the laugh, and six using the bell tone) and 16 control playbacks were conducted (six using each of the rooster and laugh, and four using the bell tone). All three sounds were played to all three groups in the test phase, although the bell tone was played to the Rita group only once and the laugh two times due to demographic changes that led to the absence of some subjects before control playbacks could be conducted (see Table 1 for the number of playbacks of each type for each group). These test and control playbacks were conducted on 18 focal individuals, including eight adult males, two adult females (≥ 4.5 years old), and eight juveniles (between 2.5 and 4.5 years old). All focal subjects had to have been present during the initial training phase. The three youngest juvenile subjects were approximately 1.5 years old (late infant/early juvenile period) during the training phase. Playbacks were conducted with the same speaker placement and equipment as used during the novel sound playbacks in the training phase. No more than one playback was conducted per group per day. The playback of the novel sound was initiated by one observer when the focal subject approached the speaker (typically at a distance of 5–12 m, although one test playback of each of the laugh and rooster crow were conducted from distances of approximately 20 and 30 m, respectively), while a second observer video recorded the reaction of the focal animal. To minimise the chances that any reactions of the focal subject were a response to the reactions of conspecifics rather than to the stimulus itself, we conducted playbacks when the focal did not have any group mates in the immediate vicinity, and when the focal subject was closer to the speaker than was any other individual (additional details below). Although we only scored reactions in the first seconds following the first playback (see below), stimuli were sometimes played a second or third time within 1 min of the initial playback to increase opportunities to further gauge responses; such multiple playbacks were conducted with both control and test stimuli, without a bias towards one or the other. At the end of the trial, a map was drawn showing the locations of the focal animal, the speaker, and the video camera, and the focal animal’s height above the ground at the initiation of the playback was noted (ranging from 1.5 to 16 m; mean ± SD 6.5 ± 4.5 m). The vast majority of these test playbacks were conducted 1–2 years after the training phase (mean ± SD number of months between the last training session and the test playback: 17.6 ± 8.4 months), although one control and one test were played 3 years after the training phase, and two controls and one test were played in the same year as the training phase (with the latter being conducted after the four training events were completed). We attempted a matched-pairs design in which each focal was played one test and one control playback (presented in random order), and were able to successfully conduct both a test and control playback with 16 subjects across the three groups; another two individuals, both juveniles in the Rita group, were observed only in the test condition because they disappeared from the group before a second playback could be conducted.

**Table 1:** The number of playbacks of each stimulus type conducted for each group, and whether the stimulus served as a test or control for that group

Group	Rooster	Laugh	Bell tone
Rita	5 (test)	2 (control)	1 (control)
Macuco	4 (control)	7 (test)	3 (control)
Spot	2 (control)	4 (control)	6 (test)

Videos of the playback trials were analysed to quantify the strength of the focal animal’s anti-predator response (vigilance, alarm calling, and escape reactions) in the first 10 s after initiation of the playback. Vigilance was defined as directing the gaze beyond the immediate substrate and within 45° of the direction of the playback speaker (as determined by the map drawn of the focal, speaker, and camera) or towards the ground. Alarm calling was defined as the production of one or more hiccup calls, a call that is given in response to terrestrial predators and is discrete from other call types in the black capuchin’s vocal repertoire (Wheeler 2010; Di Bitetti and Wheeler 2017). An escape reaction was defined as running or jumping a distance of at least 1 m. If none of these three reactions occurred, the focal animal was considered to have ignored the playback. We quantified the strength of the reaction by scoring “ignore” as a 0, “vigilance” as 1, “escape” or “alarm call” (both of which invariably also included vigilance) as 2, and a reaction comprising both “escape” and “alarm call” as 3.

All videos were coded for vigilance and escape reactions by BCW, with the sound turned off to be blind to the type of playback stimulus used, but aware of the time in which the playback occurred. Any vigilance or escape reactions, as defined above, in the 10 s following the playback were noted, along with the time they occurred. To further ensure that videos were coded reliably, 14 of the playback experiments were randomly selected and coded by BT. In this case, the videos were again coded with the sound off and blind to the playback stimulus used, but the observer was further blind to when in the video the playback occurred, noting the time of any vigilance or escape reactions occurring in a 20–35 s video clip. There was 100% agreement between the two coders in these 14 clips. Because the direction from which alarm calls came is not possible to judge from video, and because it was normally difficult from the video to visually determine whether it was indeed the focal individual that was giving alarm calls rather than another individual off camera, we relied on narration by the observer (who had the added advantage of determining whether the call came from the direction of the focal) to score whether or not and when the focal animal produced an alarm call. The videos were reviewed by BCW, however, to ensure that the calls were indeed hiccups, to determine whether or not they occurred within the 10 s timeframe, and to determine if any other individuals appeared to give an alarm prior to the narrator indicating that the focal individual did so. In all cases, the narrator’s original descriptions were confirmed by the video.

In some cases, non-focal animals engaged in conspicuous anti-predator behaviours (alarm calling, escape reactions) following the playback, but in those cases, the anti-predator reactions of the focal animal always began immediately after the playback, thus being prior to (or, at worst, simultaneous with) the reactions of conspecifics. Indeed, nearly all observed vigilance and escape reactions occurred immediately after initiation of the playback, with only a single exception in a case in which the focal did not look in the direction of the speaker until 7 s after the playback; in that case there was no other conspicuous anti-predator behaviour observed from group mates to suggest that the subject’s reaction was to conspecifics rather than the playback stimulus. Alarm vocalisations often initiated a few seconds after the stimulus was played, but in those cases, the focal animal had already engaged in vigilance and/or escape immediately following the playback, and was the first individual to give any calls.

### Statistical analysis

To test if the strength of response to a playback of a novel sound was predicted by whether the sound served as a test stimulus for that particular focal animal (i.e., the focal animal heard the sound in association with a simulated predator in the training phase) or a control stimulus (did not hear the sound in that context), a mixed-effects ordered logistic regression model was used. The dependent variable was the ordinal, four-category strength of response, while stimulus type (control or test), group membership (to control for the fact that individuals in different groups were trained with different sound stimuli), and height from the ground (to control for the possibility that focal animals close to the ground react more strongly) were included as fixed effects. Because most subjects were observed in both control and test conditions, subject ID was entered as a random effect to control for non-independence of data points (see Waller et al. 2013). To examine whether the type of sound played (i.e., rooster, laugh, bell tone) predicted the strength of response, we conducted two separate ordered logistic regressions for test and control playbacks. Here, strength of response was the dependent variable, while sound type and height from the ground were the independent variables. In the case of test playbacks, it was not necessary to run a mixed-effects model with random effects because each subject contributed only a single observation to these tests, and we were unable to include group as a random effect because this covaried completely with stimulus type. Because group and stimulus type covaried only slightly in the test of the effect of stimulus type on responses in the control conditions (*r* = 0.283), we ran a mixed-effects model with group ID included as a random effect. Finally, we used an ordered logistic regression to test whether the number of years since the training period predicted the strength of response to test stimuli, by running a model in which years since training (rounded to 0, 1, 2, or 3), playback stimulus type, and height from the ground were the independent variables. We did not run this as a mixed-effects model with subject ID or group ID as random effects because, respectively, each subject contributed only one data point and group ID covaried completely with playback stimulus type. All statistical analyses were conducted with Stat 13.0 (Stata-Corp LP, College Station, TX, USA). Data are available in the Electronic Supplementary Material (ESM).

## Results

**Fig. 2:**
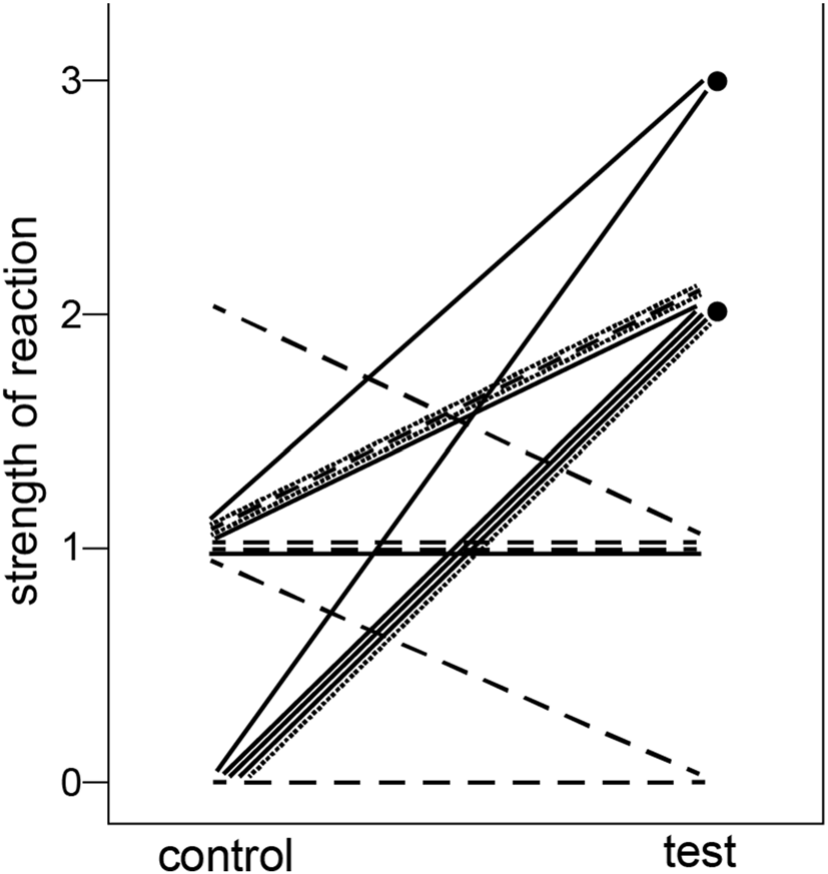
Line graph showing the strength of reaction of 18 subjects to control and test playbacks. Each line shows the matched control and test playbacks for a given individual, while the two points represent individuals for whom only a test playback was conducted (the rooster crow in both cases). Line patterns indicate the type of novel sound used in the test condition: dotted lines = rooster crow, solid lines = laugh, dashed lines = bell tone

A total of 18 test playbacks were conducted, of which two were ignored, four elicited a vigilance-only reaction, seven elicited both alarm calls and vigilance, two elicited both escape reactions and vigilance, and three elicited all three anti-predator reactions. In contrast, when these same stimulus types were played back in 16 control trials, six were ignored, nine elicited vigilance-only reactions, one elicited an escape reaction and vigilance, and none elicited alarm calls. Of the 16 individuals tested in both contexts, 10 showed a stronger reaction to the test playback than to the control, while just 2 reacted more strongly to the control, and 4 showed no difference. Both of the individuals observed only in the test but not control condition showed reactions stronger than the median reaction to controls. Stimulus type (test vs control) was found to be a significant predictor of the strength of response in the mixed-effects model (Fig. 2, Table 2).

**Table 2:** Results of the mixed-effects ordinal logistic regression testing for an effect of playback type (control vs test), group membership, and the focal animal’s height above the ground on the strength of antipredator reactions

	*β*	SE	*z*	*P*	95% CI	
Control vs test	3.115	0.878	3.550	< 0.001	1.394	4.835
Group	− 0.186	0.401	− 0.460	0.644	− 0.972	0.601
Height	− 0.169	0.087	− 1.940	0.053	− 0.340	0.002

Subject ID was included as a random effect. *N* = 34 playbacks conducted with 18 subjects

**Fig. 3:**
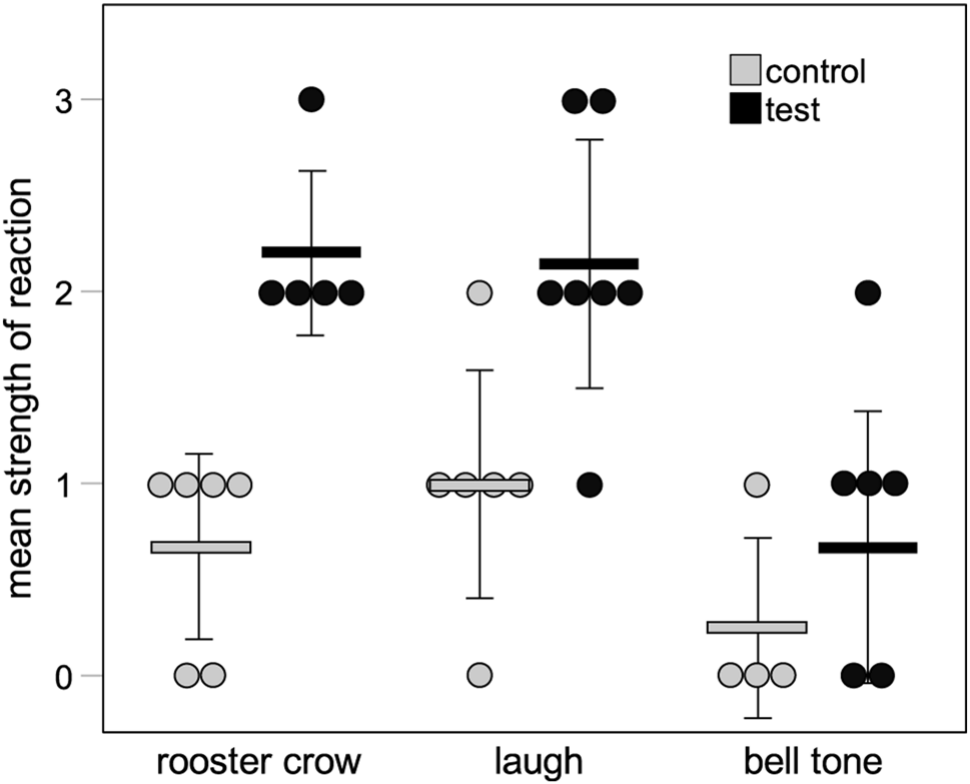
Figure showing the strength of reaction to test and control playbacks by the type of novel stimulus played. Solid bars and whiskers indicate mean ± 1 SD strength of reaction. Circles show individual data points. *N* = 18 test playbacks with 18 individuals, and 16 control playbacks with 16 of the same individuals used for the test playbacks

We then tested whether there were differences in reactions to the three novel stimuli in each of the test and control conditions. When considering the reactions to the three novel sounds when each served as a control, reactions were on average weakest in response to the bell tone and highest in response to the laugh, but the type of novel stimulus was not a significant predictor of the strength of response to these control playbacks (Fig. 3, Table 3). In contrast, when considering the reactions to the three novel sounds when each served as a test, the type of playback stimulus was a significant predictor of the strength of response of the focal animal (Fig. 3, Table 4), with reactions to the bell tone being ignored in half of the trials and eliciting a reaction stronger than vigilance-only in only one trial (an alarm call in this case). In contrast, the rooster and laugh were never ignored, and in all but three cases (one and two for the rooster and laugh, respectively) elicited alarm calls and/or escape reactions in addition to vigilance. It should be noted, however, that because test stimulus type and group ID covary completely, it cannot be determined if the observed differences in response strength are due to the stimulus type or to group differences stemming from additional factors.

**Table 3:** Results of the mixed-effects ordinal logistic regression testing for an effect of novel sound type (rooster, laugh, bell tone) and the focal animal’s height on the strength of antipredator reactions to control playbacks

	*β*	SE	*z*	*P*	95% CI	
Novel sound type	1.484	1.012	1.470	0.142	− 0.499	3.467
Height	− 0.420	0.251	− 1.670	0.095	− 0.912	0.073

Group ID included as a random effect. *N* = 16 playbacks conducted with 16 subjects from 3 groups

**Table 4:** Results of the ordinal logistic regression testing for an effect of novel sound type (rooster, laugh, bell tone) and the focal animal’s height on the strength of antipredator reactions to test playbacks

	*β*	SE	*z*	*P*	95% CI	
Novel sound type	1.916	0.794	2.410	0.016	0.360	3.472
Height	− 0.119	0.101	− 1.180	0.239	− 0.317	0.079

*N* = 18 playbacks conducted with 18 subjects

**Fig. 4:**
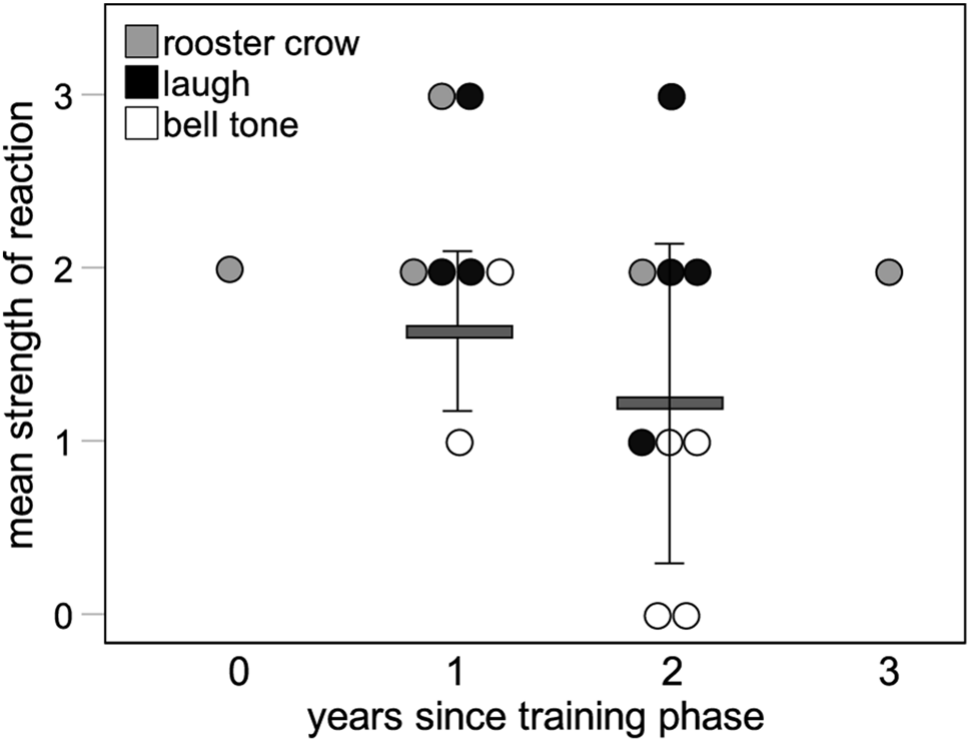
Figure showing the strength of reaction to test playbacks of the three stimulus types by the number of years since the training period. Bars and whiskers show mean ± 1 SD strength of reaction for all stimulus types in a given year. Circles show individual data points. *N* = 18 test playbacks with 18 individuals

Finally, we tested if reactions to the test stimuli weakened over time when the spatiotemporal association between the novel sounds and predators was not reinforced. Although the sample size for each year was small, the number of years since the training period was not a significant predictor of the strength of response, but there was a weak trend towards a decrease in strength of response to playbacks conducted two or more years after the training period versus those conducted earlier (Fig. 4, Table 5).

**Table 5:** Results of the ordinal logistic regression testing for an effect of the number of years since the training period, type of novel sound played back, and the focal animal’s height on the strength of antipredator reactions to test playbacks

	*β*	SE	*z*	*P*	95% CI	
Years since training	− 0.850	0.740	− 1.15	0.250	− 2.300	0.599
Novel sound type	− 1.752	0.756	− 2.32	0.021	− 3.234	− 0.269
Height	− 0.071	0.116	− 0.61	0.543	− 0.298	0.157

*N* = 18 playbacks conducted with 18 subjects

## Discussion

The results of this experiment provide strong evidence that the responses of capuchin monkeys to predator-associated sounds are driven, at least in part, by prior experience with those sounds: most individuals responded to a given sound more strongly if they previously heard that sound in association with a predator than did individuals that never heard that same sound in such a context. Although it is possible that responses to test stimuli were stronger than the controls simply because those sounds were less novel to the subjects during the test period (i.e., because they were exposed to them a greater number of times during the training phase), such interpretations conflict with empirical evidence which demonstrates that animals tend to increasingly ignore a given stimulus after repeated exposure without the co-occurrence of something salient (Cheney and Seyfarth 1988; see also; Yamaguchi et al. 2004; Leiner and Fendt 2011). Thus, when taken together with previous studies in rodents and birds (Shriner 1999; Magrath et al. 2015b), this study suggests that the widespread phenomenon of heterospecific alarm responses (Caro 2005) may typically be based on learned associations between the occurrence of such calls and the presence of predators, at least in mammals and birds if not also in other vertebrates (Ito and Mori 2010). In this light, it seems appropriate to interpret such calls as informative in the sense that they reduce uncertainty in receivers and allow them to make predictions about future events based on past associations between the signals and the presence of predators (Seyfarth et al. 2010; Fischer 2011; Wheeler and Fischer 2012).

Although it is not clear if the weaker responses to the bell tones compared to the other two sounds stems from acoustic differences between the three sound types, or if it is instead a group difference unrelated to the structural features of the test stimulus, the observed trend is in line with the contention that acoustic features typical of alarm facilitate Pavlovian learning-based fear responses in receivers (Owren and Rendall 2001). As the rooster crow and human laugh were characterised by a more noisy, broadband structure compared to the more tonal structure of the bell tone (see Fig. 1), this may explain the weakened responses to the latter in the test condition. Although suggestive, conclusions about the importance of the acoustic structure in facilitating learning in such contexts should be made with caution, as this study was based on only three sounds, and because the study design does not allow one to disentangle the effect of playback stimulus from the potential effect of group. Future studies with subjects that could be trained to associate novel sounds with aversive stimuli individually rather than in groups, such as primates in captive conditions, may be better be able to tease out the role that acoustic structure has on facilitating such learned associations using a wider range of acoustic stimuli.

The small number of sound–predator pairings necessary for capuchins to make the association, as well as the timeframe in which individuals continued to respond to their test stimuli without further reinforcement this association, was somewhat surprising, with just four sound–predator pairings being sufficient to elicit responses in subjects 9–26 months later (and in one subject 34 months later) without any subsequent reinforcement. The average strength of response did decrease slightly 2 years after the sound–predator pairings, which, together with the small sample sizes for each year, suggests that the lack of a significant difference in response strength across year should be interpreted with some caution. It should be noted, though, that additional anecdotal observations of playbacks of the laugh conducted 3 years after the final pairing, as well as a single playback of the rooster crow conducted 4 years after, elicited alarm calls from several individuals present during the training period; these are not included in the present analysis, however, because no appropriate focal animal was observed during these trials [as these were conducted as part of a follow up study to determine if individuals absent during the training phase but present during the test phase socially learned to respond to their group’s novel sound; see also Potvin et al. (2018)]. Such persisting responses in the absence of reinforcement conflicts somewhat with studies showing that, in the short term, receivers ignore calls that are less likely to reliably indicate something salient to the receiver (Cheney and Seyfarth 1988; Gouzoules et al. 1996). The absence of such habituation in the long term may be adaptive in populations in which false alarm calls are relatively common due to a “better safe than sorry” approach to unidentified phenomena (Haftorn 2000; Barnett et al. 2018), as is the case in the study population (Wheeler 2010).

In conclusion, the present study demonstrates that capuchin monkeys can learn to associate particular sounds with the presence of a predator, thus supporting the widely held contention, based largely on indirect evidence, that responses to heterospecific alarm calls are underpinned by associative learning (Magrath et al. 2015a). As such, this study provides critical support for the hypothesis that animal signals are informative for receivers (Seyfarth et al. 2010). At the same time, this study provides tentative support for the non-mutually exclusive hypothesis that signal structure is also important in driving receiver responses (Owren and Rendall 2001; Rendall et al. 2009). While it seems likely that both signal structure and associative learning shape responses to conspecific signals across a range of vertebrate and non-vertebrate taxa (Magrath et al. 2015a), further studies are needed to provide more conclusive evidence of this in a range of taxa.

## Electronic supplementary material

Below is the link to the electronic supplementary material.

Supplementary material 1 (WAV 86 kb)

Supplementary material 2 (WAV 23 kb)

Supplementary material 3 (WAV 2812 kb)

Supplementary material 4 (DOCX 181 kb)
