# Supplementary material for: Experimental evidence for heterospecific alarm signal recognition via associative learning in wild capuchin monkeys
Source: Anim Cogn. 2019 May 8;22(5):687–95. doi: 10.1007/s10071-019-01264-3 (PMC6687673; doi:10.1007/s10071-019-01264-3)
Supplement: Supplementary file 4 — Supplementary material 4 (DOCX 181 kb) [file 10071_2019_1264_MOESM4_ESM.docx]

**Electronic supplementary material for:**

**Experimental evidence for heterospecific alarm signal recognition via associative learning in wild capuchin monkeys**

**Brandon C. Wheeler^1,2,*^, Martin Fahy^3^, and Barbara Tiddi^2,4^**

^1^School of Anthropology and Conservation, University of Kent, Canterbury CT2 7NR, United Kingdom

^2^Cognitive Ethology Laboratory, German Primate Center, 37077 Göttingen, Germany

^3^Proyecto Caí, Iguazú National Park, Misiones, Argentina

^4^Department of Behavioral Ecology, Johann-Friedrich-Blumenbach Institute for Zoology and Anthropology, Georg-August University, 37077 Göttingen, Germany

*Corresponding author (bcwheeler43@gmail.com)

Details on decoy ocelot models

During the training period, capuchins were exposed to a novel sound together with a simulated predator, including either a decoy ocelot (*Leopardus pardalis*) model or a playback of a recording of a vocalising puma (*Puma concolor*). The decoy ocelot was constructed by mounting a faux-ocelot fur coat on a taxidermist’s bobcat (*Lynx rufus*) manikin (See Fig. S1).


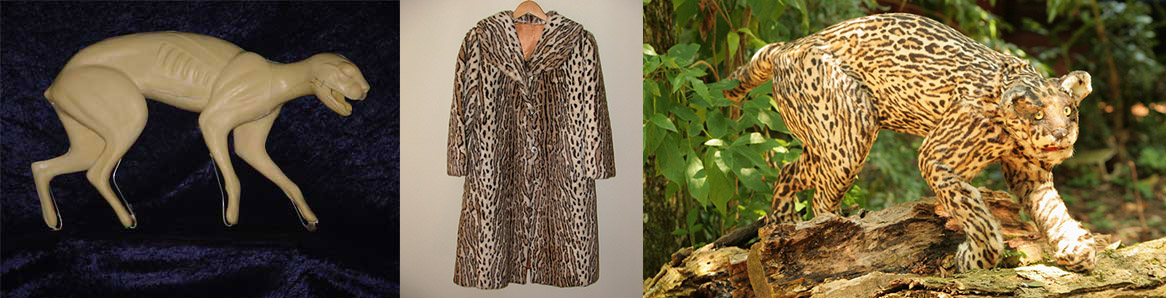


**Figure S1.** The bobcat manikin (left) and faux fur coat (center) used to construct the model, and the model used in the current study (right).

| date of playback | focal ID | focal age | focal sex | focal group | playback stimulus | type | height (m) | vigilance? | alarm? | escape? | | total strength of reaction | years since training phase | months since training phase |
| --- | --- | --- | --- | --- | --- | --- | --- | --- | --- | --- | --- | --- | --- | --- |
| 08/07/2012 | AST | juvenile | male | Macuco | bell tone | control | 4.0 | no | no | | no | 0 |  |  |
| 17/07/2013 | AST | juvenile | male | Macuco | laugh | test | 2.0 | yes | yes | | yes | 3 | 1 | 10.9 |
| 21/05/2013 | DAL | adult | male | Macuco | bell tone | control | 1.5 | no | no | | no | 0 |  |  |
| 06/07/2013 | DAL | adult | male | Macuco | laugh | test | 2.5 | yes | no | | yes | 2 | 1 | 10.5 |
| 20/06/2014 | EDU | adult | male | Macuco | laugh | test | 4.0 | yes | yes | | yes | 3 | 2 | 22.0 |
| 20/07/2014 | EDU | adult | male | Macuco | rooster | control | 8.0 | yes | no | | no | 1 |  |  |
| 16/07/2013 | ERN | adult | male | Macuco | laugh | test | 3.0 | yes | yes | | no | 2 | 1 | 10.8 |
| 24/08/2011 | ERN | adult | male | Macuco | rooster | control | 7.0 | no | no | | no | 0 |  |  |
| 01/08/2014 | MAW | adult | female | Macuco | laugh | test | 16.0 | yes | no | | no | 1 | 2 | 23.4 |
| 05/08/2014 | MAW | adult | female | Macuco | rooster | control | 3.0 | yes | no | | no | 1 |  |  |
| 21/07/2014 | MOR | juvenile | female | Macuco | laugh | test | 8.0 | yes | yes | | no | 2 | 2 | 23.0 |
| 29/07/2014 | MOR | juvenile | female | Macuco | rooster | control | 1.5 | yes | no | | no | 1 |  |  |
| 26/07/2014 | RIC | adult | male | Macuco | bell tone | control | 10.0 | no | no | | no | 0 |  |  |
| 04/08/2014 | RIC | adult | male | Macuco | laugh | test | 5.0 | yes | yes | | no | 2 | 2 | 23.5 |
| 15/06/2014 | HOR | adult | male | Rita | laugh | control | 4.5 | no | no | | no | 0 |  |  |
| 19/07/2012 | HOR | adult | male | Rita | rooster | test | 6.0 | yes | yes | | no | 2 | 1 | 11.0 |
| 08/08/2014 | ILA | juvenile | female | Rita | bell tone | control | 4.0 | yes | no | | no | 1 |  |  |
| 20/06/2014 | ILA | juvenile | female | Rita | rooster | test | 3.0 | yes | yes | | no | 2 | 3 | 34.0 |
| 24/08/2011 | JAC | juvenile | male | Rita | rooster | test | 8.0 | yes | no | | yes | 2 | 0 | 0.1 |
| 09/08/2011 | MAR | adult | male | Rita | laugh | control | 4.0 | yes | no | | no | 1 |  |  |
| 18/07/2013 | MAR | adult | male | Rita | rooster | test | 6.0 | yes | yes | | no | 2 | 2 | 23.0 |
| 25/07/2012 | SEA | juvenile | male | Rita | rooster | test | 15.0 | yes | yes | | yes | 3 | 1 | 11.2 |
| 06/07/2013 | CAM | juvenile | male | Spot | bell tone | test | 5.0 | yes | yes | | no | 2 | 1 | 10.6 |
| 17/07/2013 | CAM | juvenile | male | Spot | laugh | control | 6.0 | yes | no | | no | 1 |  |  |
| 18/07/2014 | FRA | adult | female | Spot | bell tone | test | 15.0 | yes | no | | no | 1 | 2 | 23.0 |
| 27/06/2013 | FRA | adult | female | Spot | laugh | control | 2.5 | yes | no | | no | 1 |  |  |
| 25/07/2014 | RIN | adult | male | Spot | bell tone | test | 15.0 | yes | no | | no | 1 | 2 | 23.2 |
| 21/07/2014 | RIN | adult | male | Spot | laugh | control | 4.0 | yes | no | | yes | 2 |  |  |
| 22/05/2013 | ROB | juvenile | male | Spot | bell tone | test | 2.0 | no | no | | no | 1 | 1 | 9.1 |
| 15/07/2013 | ROB | juvenile | male | Spot | laugh | control | 2.5 | yes | no | | no | 1 |  |  |
| 15/08/2014 | SPA | juvenile | male | Spot | bell tone | test | 7.0 | no | no | | no | 0 | 2 | 23.9 |
| 01/08/2014 | SPA | juvenile | male | Spot | rooster | control | 16.0 | no | no | | no | 0 |  |  |
| 31/07/2014 | TRU | adult | male | Spot | bell tone | test | 10.0 | no | no | | no | 0 | 2 | 23.4 |
| 14/08/2014 | TRU | adult | male | Spot | rooster | control | 10.0 | yes | no | | no | 1 |  |  |
